# Gut Microbiota of Individuals Could Be Balanced by a 14-Day Supplementation With *Laminaria japonica* and Differed in Metabolizing Alginate and Galactofucan

**DOI:** 10.3389/fnut.2022.881464

**Published:** 2022-05-18

**Authors:** Xueqian Zhang, Changyu Su, Cui Cao, Guiping Gong, Linjuan Huang, Zhongfu Wang, Shuang Song, Beiwei Zhu

**Affiliations:** ^1^College of Food Science and Technology, Northwest University, Xi'an, China; ^2^National Engineering Research Center of Seafood, School of Food Science and Technology, Dalian Polytechnic University, Dalian, China; ^3^Shaanxi Natural Carbohydrate Resource Engineering Research Center, College of Food Science and Technology, Northwest University, Xi'an, China; ^4^National & Local Joint Engineering Laboratory for Marine Bioactive Polysaccharide Development and Application, Dalian Polytechnic University, Dalian, China

**Keywords:** *Laminaria japonica*, polysaccharide, fermentation, alginate, fucoidan, gut microbiota

## Abstract

*Laminaria japonica* is rich in alginate (Alg) and galactofucan (GF) which have both been reported to regulate gut microbiota composition. To reveal the effect of *L. japonica* on human gut microbiota, the fecal microbiota of 12 volunteers before and after 14-day *L. japonica* intake was sequenced and compared, and the capabilities of the gut microbiota to utilize Alg and GF were also investigated. The 16S rRNA gene sequencing results demonstrated that *Firmicutes/Bacteroidetes* ratio could be balanced by *L. japonica* supplementation. The ability of gut microbiota to utilize Alg was significantly enhanced by *L. japonica* supplementation. Furthermore, the multiple linear regression analysis suggested that bacteria from *Bacteroidaceae* and *Ruminococcaceae* were positively correlated with Alg utilization while those from *Erysipelotrichaceae, Bacteroidaceae*, and *Prevotellaceae* participated in GF degradation. Moreover, the production of acetic acid and the total short-chain fatty acids (SCFAs) in fermentation were consistent with the consumption of Alg or GF, and propionic acid content was positively correlated with Alg consumption. In addition, the percentage of monosaccharides in the consumed GF after the fermentation suggested that gut microbiota from individuals could consume GF with different monosaccharide preferences. These findings shed a light on the impacts of dietary *L. japonica* on human health.

## Introduction

*Laminaria japonica*, one of the most widely consumed commercial edible seaweeds in the world (1, 2), has a high production in China, Japan, and Korea (3–5). It is rich in dietary fiber, such as alginate (Alg) and fucoidan, and their contents generally vary from 30 to 40% and 5 to 10% of the seaweed dry weight, respectively (6). Alg is an acidic polysaccharide composed of 1,4-linked β-D-mannuronate (M) and 1,4-linked α-L-guluronate (G) (7). Fucoidan is a class of sulfated polysaccharides in brown algae containing fucose and varying greatly in their structural characteristics (8). Galactofucan (GF) found in *Laminaria japonica* is a type of fucoidan, consisting of fucose and galactose residues (9).

Both Alg and fucoidan are indigestible fibers that could modulate the composition and/or activity of microorganisms in the large intestine, thus conferring a beneficial physiological effect on the host (10). For instance, Alg could prevent high-fat diet-induced metabolic syndrome and increase the abundance of *Bacteroides* (11), and relieve hyperglycemia in type 2 diabetic mice by enriching *Bacteroides, Lactobacillus, Akkermansia, Alloprevotella, Weissella*, and *Enterorhabdus*, and decreasing *Turicibacter* and *Helicobacter* in the intestinal tracts of mice (12). Furthermore, fucoidan shows a therapeutic effect on metabolic syndrome and the improvement of gut dysbiosis in association with an increase of *Akkermansia* population in the gut microbes of high-fat diet mice (13). More recently, researchers have suggested that fucoidan could improve insulin resistance in diet-induced obese mice by remodeling gut microbiota (14). It could be concluded that both Alg and fucoidan could modulate gut microbiota in animal models, suggesting the benefits of *L. japonica* consumption on the ecosystem of the intestinal tract. Therefore, it is necessary to evaluate the effect of *L*. *japonica* in human dietary intervention studies. In addition, more and more pieces of evidence demonstrate that the utilization of dietary fibers by gut microbiota could produce short-chain fatty acids (SCFAs) and other metabolites to benefit the host. However, bacteria responsible for the breakdown and utilization of Alg and fucoidan are largely unknown.

The present study aimed to evaluate the effects of dietary *L. japonica* supplementation on the gut microbiota of normal humans and the capabilities of the gut microbiota to utilize Alg and GF, and reveal bacteria involved in the utilization of Alg and GF. The microbial communities from twelve volunteers before and after the 14-day supplementation with *L. japonica* were compared. Furthermore, the fermentation of Alg and GF from *L. japonica* and SCFAs production by these fecal microbiota communities from different individuals were assessed.

## Materials and Methods

### Materials

Instant shredded kelp was provided by Sichuan Jixiangju Food Co., Ltd. (Sichuan, China). Dried *L. japonica* was obtained from Fujian Yida Food Co., Ltd. (Fujian, China). Alg was purchased from Qingdao Bright Moon Seaweed Group Co., Ltd. Its molecular weight was determined as 1,311 kDa according to the reported method (15). GF was prepared from *L. japonica* according to our previous method and its monosaccharide composition was fucose (Fuc): xylose (Xyl): galactose (Gal): glucose (Glc): galacturonic acid (GalA): manmose (Man) = 9.8: 1.0: 16.4: 1.8: 6.0: 12.7 as reported by us previously (15), and the average molecular weight of GF was determined as 627 kDa. Peptone and yeast extract were purchased from Oxoid (Thermo Co., Ltd, Massachusetts, USA). Monosaccharide standards, such as rhamnose (Rha), Xyl, Gal, GalA, Glc, glucuronic acid (GlcA), Man, arabinose (Ara), and Fuc were purchased from Sigma Chemical Co. (St. Louis, MO, USA). The ingredients of bacterial media were purchased from Qingdao Hope Bio-Technology Co., Ltd. All other chemical reagents with analytical grades used in this study were purchased from Sinopharm Chemical Reagent Co., Ltd. (Shanghai, China).

### Subjects and Diet

In this study, 12 subjects were chosen from six male and six female volunteer students (aged 20–22 years old) of the School of Food Science and Technology, Dalian Polytechnic University. The volunteers consumed *L. japonica* 50 g/day for 14 days. The participants had no gastrointestinal disease or antibiotics treatment for at least 3 months. The study was supported by the Ethics Committee of Dalian Polytechnic University and all volunteers provided a well-informed and signed consent before entering the trial. The general characteristics of the 12 individuals during intervention are shown in Supplementary Table S1. The body mass index (BMI) of volunteers was 18.5–23.9 which is in the healthy weight range. The volunteers were asked not to take any medication that could affect the gastrointestinal tract during the trial.

### High-Throughput Sequencing Analysis

The fecal samples obtained from the 12 normal volunteers before (Be) and after (Af) 14-day dietary intake of *L. japonica* were labeled as Be.1, Be.2, Be.3 to Be.12 and Af.1, Af.2, Af.3 to Af.12, respectively. The PowerFecal™ DNA Isolation Kit (MO BIO, USA) was applied to isolate the genomic DNA from the feces, and the analysis of all the DNA samples was conducted by Novogene Bioinformatics Technology Co., Ltd. (Beijing, China). For each sample, the V3-V4 region of 16S rRNA was chosen for amplification using the forward primer 515F and the reverse primer 806R and sequenced by Ion S5TMXL platform. The analysis was based on sequenced reads and operational taxonomic units (OTUs).

### *In vitro* Fermentation of Alg and GF

#### Preparation of Growth Medium

The preparation of growth medium was performed according to the method reported previously (16). Briefly, 1 L of the basal nutrient medium was composed of 2 g peptone, 2 g yeast extract, 0.02 g hemin, 0.5 g L -cysteine, 0.5 g bile salts, 0.1 g NaCl, 0.04 g K_2_HPO_4_, 0.04 g KH_2_PO_4_, 0.01 g MgSO_4_·7H_2_O, 0.01 g CaCl_2_·6H_2_O, 2 g NaHCO_3_, 1 mg resazurin, 2 ml Tween-80, and 10 μl vitamin K. The whole substrates were dissolved in distilled water and autoclaved at 121°C for 15 min.

#### Preparation of Human Fecal Slurry and *in vitro* Fermentation

A total of 24 fecal samples as mentioned in Section High-Throughput Sequencing Analysis were used *in vitro* fermentation. Then, 1 g of feces from the donor was diluted in a 9-ml modified physiological saline solution containing 9 g/L of NaCl and blended using a magnetic stirrer to yield 10% (w/v) fecal slurry. Then, the fecal slurry was centrifuged at 4°C (500 rpm, 5 min). After that, 1.0 ml of the fecal suspension was added to 9.0 ml of the basal nutrient medium containing 3 g/L of Alg or 3 g/L of GF. All samples were incubated at 37°C in an anaerobic incubator (Electrotek, UK), and 1.5 ml fermentation samples were collected at 0 and 48 h and then stored at −80°C for further analysis. Each experiment was independently replicated 3 times.

### Measurement of Residual Carbohydrates

The results were expressed as the proportion of residual Alg or GF (%) based on the contents of carbohydrates before and after fermentation. Residual Alg content was measured by using the *meta*-hydroxydiphenyl colorimetric method with Alg as standard (17) and residual GF content was determined using the phenol-sulfuric acid method with Gal as standard (18), respectively.

### Determination of Contents of SCFAs

The contents of SCFAs, such as acetic acid, propionic acid, and butyric acid were measured according to the procedure reported with some modifications (19). Briefly, 500 μl of the fermentation broth was transferred into a 2-ml centrifuge tube, and the solution was acidified by adding 10 μl of sulfuric acid (50%, v/v). Subsequently, 900 μl of diethyl ether and 100 μl of 500 μg/ml 2-ethylbutyric acid (internal standard) solution were added to the sample. The solution was then mixed through vortex oscillation 2 times for 3 min. After being centrifuged (12,000 rpm, 15 min), the supernatant was transferred into a tube with 250 mg of Na_2_SO_4_. The mixed solution was centrifuged at 4°C and the supernatant was loaded by the GC (2010-plus, Shimadzu) equipped with a flame ionization detector (FID), and a Rtx®-Wax column (30 m × 0.25 mm × 0.25 μm) was used. For GC analysis, the initial column temperature was 100°C with a 1-min hold, then programmed to 180°C at a temperature ramp of 5°C/min and maintained for 4 min; the injector temperature was 250°C; the flow rates of hydrogen, air, and nitrogen make up gas were 40, 300, and 3 ml/min, respectively.

### Analysis of Monosaccharide Composition

The monosaccharide composition analysis of GF after 48 h fermentation was performed as the previous method with a little modification (20). In brief, after dialyzed and dried, the sample was dissolved in 2 ml of 2 M trifluoroacetic acid (TFA) at 121°C for 2 h. Then, excess TFA was removed with deionized water by reduced pressure distillation. The residue was re-dissolved in 500 μl of Na_2_CO_3_ (0.5 mol/L) solution and maintained at 30°C for 45 min. After the reduction using sodium borohydride, acetic acid was added to the solution. Sodium in the obtained solution was removed by passing through a cation exchange column. Excess borohydride was decomposed by co-distillation with methanol. The resultant solution was heated at 85°C for 2 h to convert the aldose salt to lactone. The reagent of pyridine and n-propylamine (1 ml each) were added to dissolve the residue and then kept at 55°C for 30 min to react. Then, the solution was dried in vacuum. After adding 1 ml pyridine and 1 ml acetic anhydride, the solution was kept at room temperature overnight. The resulting solution was extracted 3 times using dichloromethane. Neutral sugars and uronic acids were subsequently determined by GC using the method reported previously (21).

### Statistic Analysis

A multivariate linear regression model was used to investigate the associations between Alg or GF utilization and bacterial OTUs. The relative abundances of bacterial OTUs with an abundance larger than 5% in at least one sample were included in the regression model as confounding factors. Data were analyzed using SPSS version 20.0 software (SPSS Inc., Chicago, IL, USA). All the data were presented as means ± standard derivation (SD). Comparison between two groups/samples was performed using Student's *t*-test. The comparison of multiple samples was conducted by one-way analysis of variance (ANOVA) procedure followed by the Tukey's Honestly Significant Difference (HSD) test. Correlations were analyzed using Pearson's correlation. The values of *p* < 0.05 were regarded as statistically significant. ^*^p < 0.05, ^**^p < 0.01, and ^***^p < 0.001.

## Results and Discussion

### Effects of *L. Japonica* Supplementation on Normal Human Gut Microbiota Structures

**Figure 1:**
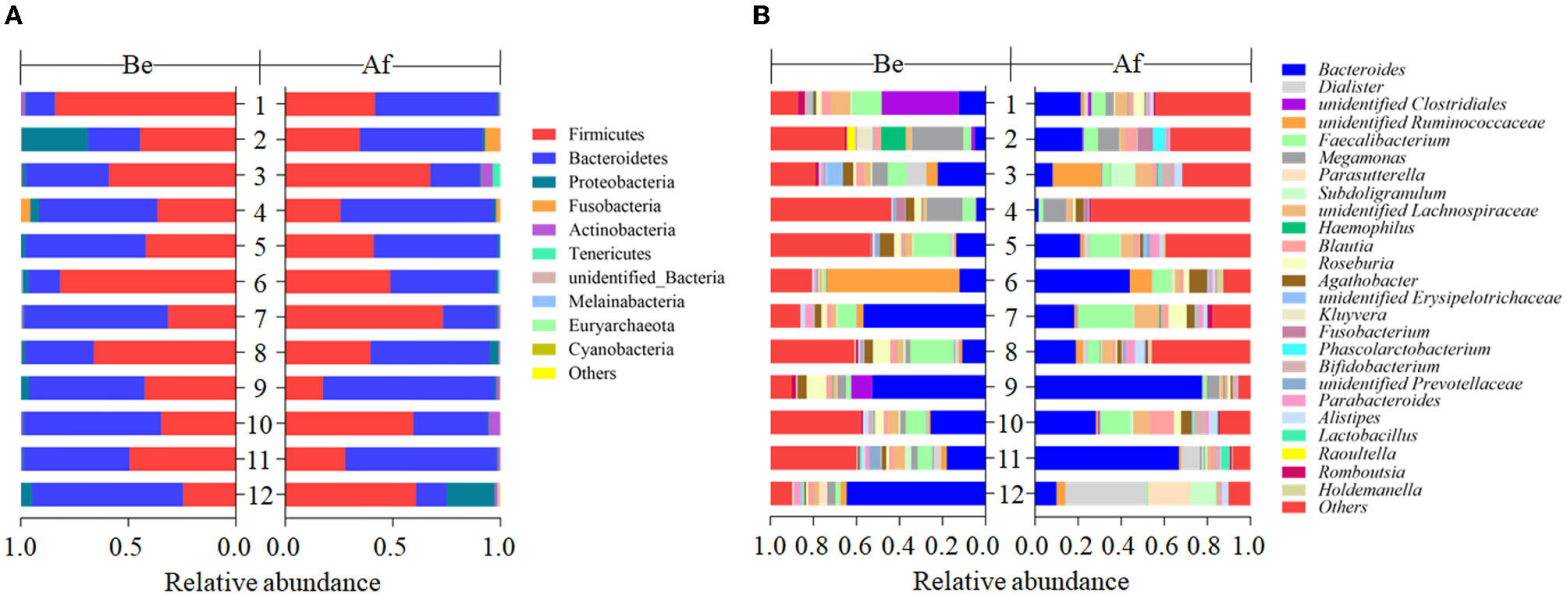
The compositions of fecal gut microbiota before (Be) and after (Af) the 14-day dietary *Laminaria japonica* intake at the phylum level **(A)** and genus level **(B)**.

**Figure 2:**
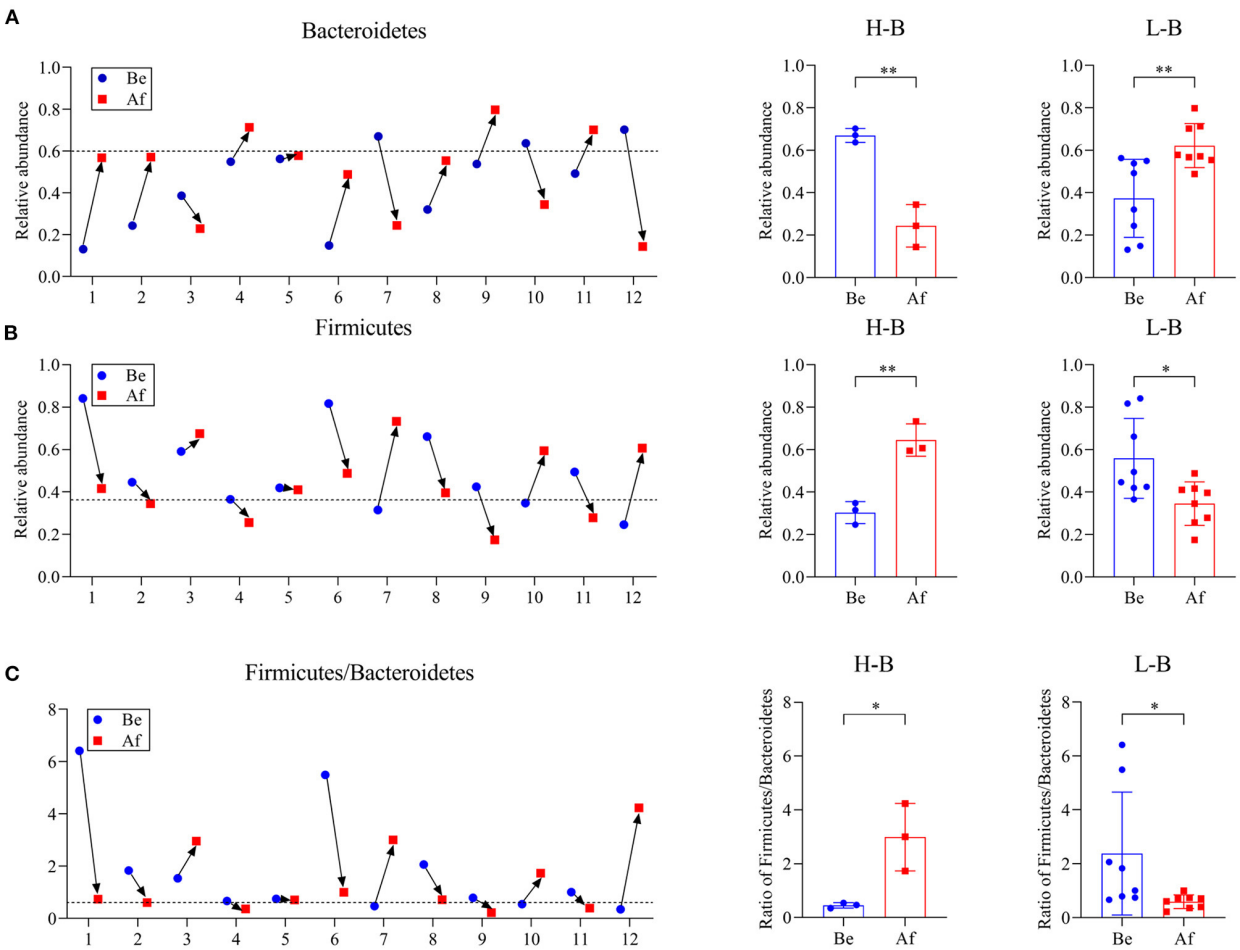
The phylum *Bacteroidetes* abundance **(A)**, phylum *Firmicutes* abundance **(B)**, and *Firmicute/Bacteroidetes* ratio **(C)** in individuals Be and Af the 14-day dietary *L. japonica* intake and the comparations of microbiota abundance in the H-B group and the L-B group. H-B group, original *Bacteroidetes* abundance higher than 0.6; L-B group, original Bacteroidetes abundance lower than 0.6. *p < 0.05 and **p < 0.01.

The effects of a 14-day supplementation with *L. japonica* on gut microbiota structures were evaluated by comparing the gut microbes of the volunteers before (Be group) and after (Af group) the dietary intervention. The gut microbiota compositions in most of the individuals varied obviously after supplementation with *L. japonica* both at phylum (Figure 1A) and genus (Figure 1B) levels. Then, the changes of *Bacteroidetes* and *Firmicutes* were observed at the individual level. The change directions of *Bacteroidetes* (Figure 2A) and *Firmicutes* (Figure 2B) in these 12 individuals were not consistent. However, interestingly, those with the original *Bacteroidetes* abundance higher than 0.6 (Nos. 7, 10, and 12), grouped as H-B, all down-adjusted the *Bacteroidetes* after the intake of *L. japonica* while those with *Bacteroidetes* lower than 0.6 (Nos. 1, 2, 4, 5, 6, 8, 9, and 11), grouped as L-B, demonstrated an opposite trend, except No. 3. Further significance analysis excluding No. 3 indicated that *L. japonica* intake could significantly reduce *Bacteroidetes* in the H-B group but increase *Bacteroidetes* in the L-B group. Moreover, the original Firmicutes abundance of more than 0.36 showed a reduced population after the intervention of *L. japonica*. Further analysis demonstrated that the modulation of *Firmicutes* in the H-B group and the L-B group was opposite to *Bacteroidetes*. Similar patterns were observed in *Firmicures/Bacteroidetes* (divided based on the line at 0.6) (Figure 2C). Previous studies have established the correlation of an increase in the *Firmicutes*-to-*Bacteroidetes* ratio to the higher energy harvesting from diet and the pathology of obesity (22–24). Dietary fiber was effective to downregulate the *Firmicutes*-to-*Bacteroidetes* ratio in obese patients and thus attenuated obesity (25–28). However, in the model of dextran sulfate sodium (DSS)-induced inflammatory mice, supplementation with polysaccharides could upregulate the *Firmicutes*-to-*Bacteroidetes* ratio and alleviate intestinal inflammation (29). In the present study, *L. japonica* supplementation balanced the population of *Firmicutes* to *Bacteriodetes* ratio in individuals, which may be associated with the intake of dietary fiber in *L. japonica*. The different changed directions of the microbiota abundance between the H-B group and the L-B group may be explained by the variation among inter-individuals.

### Effects of *L. Japonica* Supplementation on the Capabilities of Fecal Microbiota to Metabolize Alg and GF

**Figure 3:**
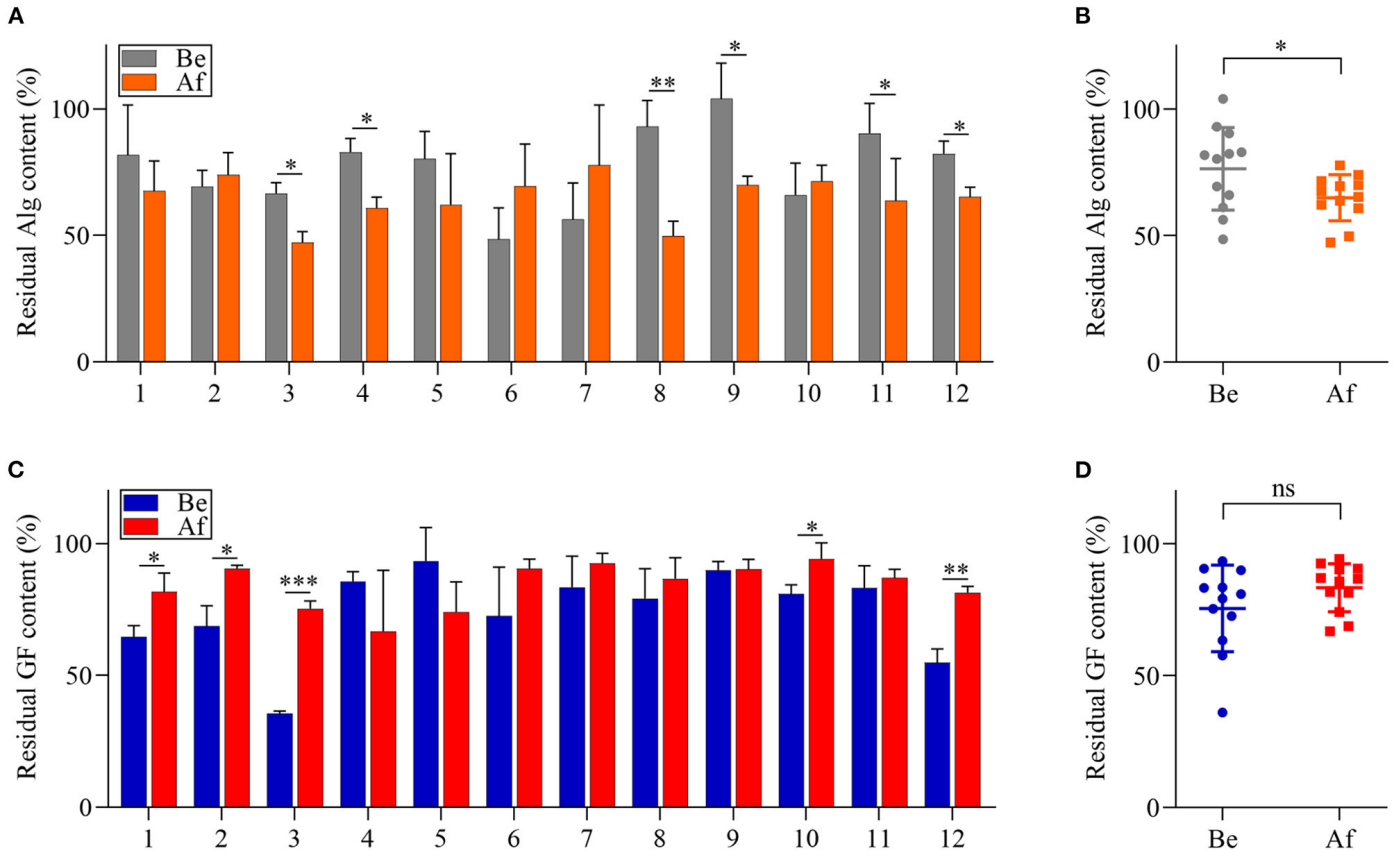
The comparisons of alginate (Alg) and galactofucan (GF) utilization by fecal microbiota collected Be and Af *L. japonica* intake in individuals **(A,C)** and in groups **(B,D)**. *p < 0.05, **p < 0.01, ***p < 0.001; ns, no significant difference.

The utilization of Alg and GF by the fecal microbiota of 12 volunteers before and after the 14-day *L. japonica* intake was evaluated by determining the remaining sugar contents after 48 h of fermentation. As shown in Figure 3A, the residual content of Alg differed greatly among the samples in both the Be group and the Af group. However, for one-half of the participants, the Alg content after fermentation in the Af group was significantly lower than that in the Be group. Moreover, Figure 3B showed that there was a significant difference between the Be group and the Af group in residual Alg content, indicating that *L. japonica* intake could enhance the capability of fecal microbiota to metabolize Alg. Unlike Alg consumption, the GF content after fermentation in the Af group did not show more utilization by fecal microbiota than that in the Be group (Figure 3C) and no significant change in the GF utilization between the Be group and the Af group was observed (Figure 3D). It has been well documented that some dietary fibers could regulate the gut microbiota community when these microbiota consuming fibers. As the major component of *L. japonica*, Alg (30–40% of dry weight) (30) was supposed to show a far more important effect on gut microbiota than GF. Moreover, the degradation rates of Alg and GF by gut microbiota differed because of their different chemical structures, and obviously, Alg was favored more by gut microbiota than GF. Of note, *L. japonica* intake enhanced the Alg fermentation ability of fecal bacteria in Nos. 3, 4, 8, 9, 11, and 12. Of the individuals with the greatest consumption of Alg, Nos. 4, 8, 9, and 11 showed increased *Bacteroidetes* but No. 3 and 12 showed a reduction in *Bacteroidetes* (Figure 2A). Altogether, these results implied the discrepancy of Alg or GF utilization efficacy among individuals after *L. japonica* intake. This finding was consistent with the previous report that the fecal microbiota from different individuals differed in their ability to utilize polysaccharides (31). Previous studies have demonstrated that chondroitin sulfate could be readily metabolized to varying extents by diverse microbial consortiums (32). Recent studies revealed that the regulation results of gut microbiota by resistant starch varied greatly due to inter-individual differences in gut microbiota populations (33). These studies suggest that the beneficial outcomes of polysaccharides are influenced by the inter-individual variation of microbiome structure.

### Gut Microbiota Involved in the Fermentation of Alg and GF

The multiple linear regression analysis is a regression model with more than one independent variable which is applied to determine the correlations among variables having cause-effect relationships and to make predictions for the topic by using the relationships (31, 34, 35). To identify the determinants of bacterial OTUs to Alg or GF utilization, the association of fecal bacterial OTUs with the consumption of the Alg or GF was analyzed by multiple linear regression analysis. The results are shown in Table 1, and the multiple linear regression equation was obtained as follows: the consumption of Alg = 0.356–4.450OTU_18_ + 3.841OTU_200_-3.222OTU_75_ + 1.064OTU_28_ + 0.288OTU_7_, with a correlation coefficient of 0.908, and the consumption of GF = 0.177 + 7.744OTU_66_ + 5.714OTU_1060_ + 3.774OTU_589_-2.565OTU_18_, with a correlation coefficient of 0.798. Totally, 5 OTUs and 4 OTUs were the significant variables attributed to Alg and GF utilization, respectively. The multiple linear regression analysis showed that the consumption of Alg was positively related (B > 0) with *Bacteroide smassiliensis* (OTU_200_), *Ruminococcus bromii* (OTU_28_), and a genus of *Ruminococcaceae* (OTU_7_), and negatively related (B < 0) with OTU_18_ (*Roseburia inulinivorans*) and a genus of *Muribaculaceae* (OTU_75_). The consumption of GF had a positive correlation with a genus of *Erysipelotrichaceae* (OTU_66_), *Bacteroides dorei* (OTU_1060_), and a genus of *Prevotellaceae* (OTU_589_), and a negative correlation with *R. inulinivorans* (OTU_18_). *Bacteroides massiliensis* and a genus of *Erysipelotrichaceae* (OTU_66_) were the most powerful bacteria to metabolize Alg and GF, respectively. In addition, a multi-collinearity analysis demonstrated no interaction among these variables (tolerance > 0.1). *Bacteroidaceae* and *Prevotellaceae*, which are reported as the major gut microbiota families participating in the degradation of numerous polysaccharides (36–38), also play important roles in the utilization of Alg and GF. Moreover, it has been reported that most of the Alg-active bacteria are from *Bacteroides* (39), and *Bacteroides* could also be enriched by fucoidan (13), suggesting their possible participation in GF degradation in the gut. In addition, the members of the *Ruminococcaceae* family could also take part in the Alg utilization, as indicated by its potential power to degrade indigestible polysaccharides (40, 41). *Erysipelotrichaceae*, which is involved in the fermentation of plant polysaccharides (42), also demonstrated its attribution in GF consumption. Nevertheless, although *Roseburia* and *Muribaculaceae* are associated with carbohydrate metabolism (43, 44), they had an unexpected negative association with the consumption of Alg and/or GF. Notably, the bacteria members contributing to the degradation of Alg and GF were discrepant, indicating that different carbohydrate-active enzymes (CAZymes) were involved in the metabolism of the 2 polysaccharides. The inhomogeneity of the regulation on these microbiotas by *L. japonica* supplementation suggested the bacteria members participating in the polysaccharide degradation varied in individuals.

**Table 1:** A multiple linear regression analysis between bacterial OTUs (%) and alginate (Alg) or galactofucan (GF) consumption (%).

	**Variable**	**B**	***p***	**Tolerance**	**Family**	**Species**
Alg	Intercept	0.356	0.000			
(R = 0.908)	OTU_18_	−4.450	0.000	0.841	Lachnospiraceae	*Roseburia inulinivorans*
	OTU_200_	3.841	0.000	0.899	Bacteroidaceae	*Bacteroides massiliensis*
	OTU_75_	−3.222	0.004	0.960	Muribaculaceae	*Unidentified*
	OTU_28_	1.064	0.013	0.947	Ruminococcaceae	*Ruminococcus bromii*
	OTU_7_	0.288	0.043	0.958	Ruminococcaceae	*Unidentified*
GF	Intercept	0.177	0.011			
(R = 0.798)	OTU_66_	7.744	0.001	0.972	Erysipelotrichaceae	*Unidentified*
	OTU_1060_	5.714	0.010	0.967	Bacteroidaceae	*Bacteroides dorei*
	OTU_589_	3.774	0.019	0.969	Prevotellaceae	*Unidentified*
	OTU_18_	−2.565	0.044	0.990	Lachnospiraceae	*Roseburia inulinivorans*

*R, correlation coefficient; B, unstandardized coefficients*.

### SCFAs Production in the Fermentation of Alg and GF

**Figure 4:**
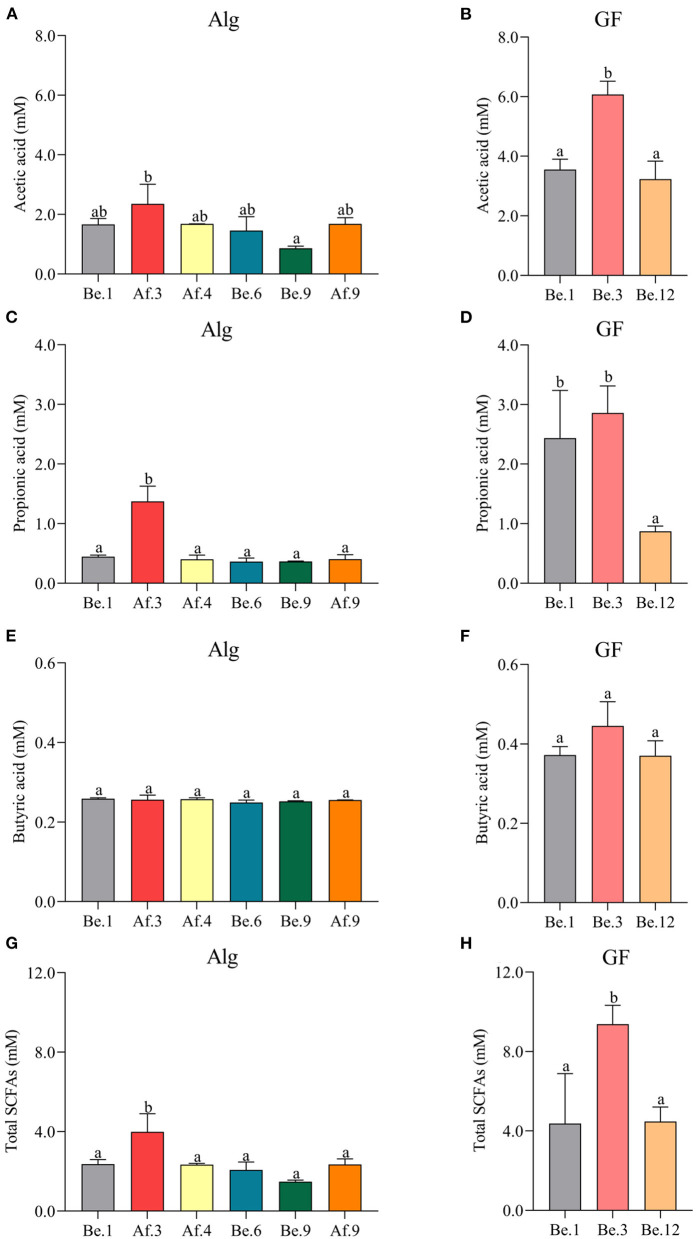
The productions of acetic acid **(A,B)**, propionic acid **(C,D)**, butyric acid **(E,F)**, and total short-chain fatty acids (SCFAs) **(G,H)** after the fermentation of Alg and GF for 48 h. Graph bars marked with different letters on top represent statistically significant results (*p* < 0.05) based on one-way analysis of variance (ANOVA) followed by the Tukey's Honestly Significant Difference (HSD) test, whereas bars labeled with the same letter correspond to results that show no statistically significant differences.

**Figure 5:**
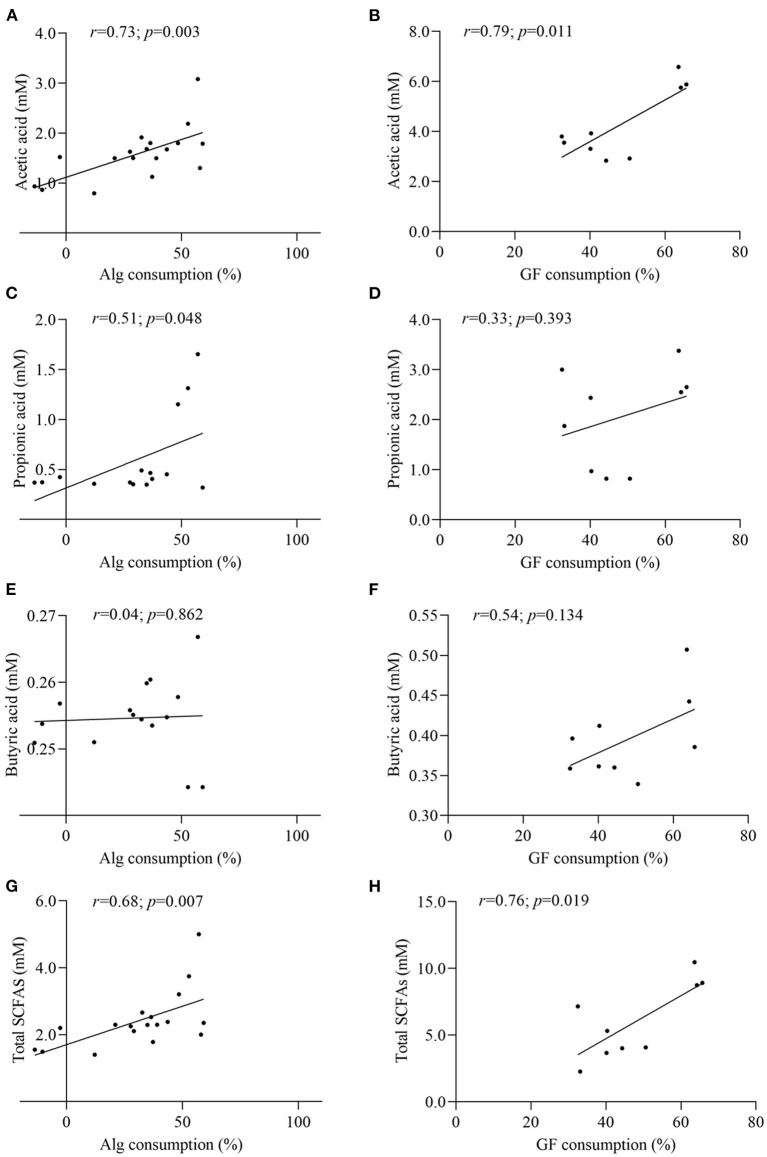
Correlation analysis between Alg and GF consumption with the contents of acetic acid **(A,B)**, propionic acid **(C,D)**, butyric acid **(E,F)**, and total SCFAs **(G,H)**.

In the light of the fact that gut microbiota variation will lead to the distinct metabolic functions for Alg and GF, SCFAs, the most important microbial metabolites of dietary polysaccharides, produced by human microbiota from different individuals were compared and the correlation analysis between SCFAs and the Alg or GF consumption was further conducted. Fecal samples with the highest (Af.3) or lowest (Be.9) Alg utilization capabilities and those with more (Af.4 and Af.9) or less (Be.1 and Be.6) *Bacteroidetes* were chosen for the assay of SCFAs production. As shown in Figures 4A,B, among these samples, Af.3 and Be.3 produced the most acetic acid in the fermentation of Alg and GF, respectively, and of note, they were the most potent to degrade Alg and GF (as shown in Section Effects of *L. Japonica* Supplementation on the Capabilities of Fecal Microbiota to Metabolize Alg and GF), respectively. Moreover, Af.3 and Be.3 showed a comparatively high production of propionic acid in Alg and GF fermentation, respectively (Figures 4C,D). However, no significant differences among individuals were observed in the butyric acid concentration for both Alg and GF fermentation (Figures 4E,F). In addition, Af.3 and Be.3, which afforded the most acetic acid, produced the highest amounts of the total SCFAs in the fermentation of Alg and GF, respectively (Figures 4G,H). Subsequently, the correlation analysis showed that the production of the acetic acid and the total SCFAs were positively correlated with the consumption of both Alg and GF (Figure 5), and the production of propionic acid was positively associated with the utilization of Alg. It is well known that acetic acid and propionic acid are both beneficial for host health (45). For instance, acetic acid can serve as an energy source for gut microbiota (46) and peripheral tissues (47). Propionic acid can be converted into glucose by intestinal gluconeogenesis, leading to satiety and decreased hepatic glucose production (48). The differences in the productions of SCFAs among the fecal samples were related to the abilities of the gut microbiota to degrade Alg or GF, and SCFAs production was greatly correlated with Alg or GF utilization. Considering the important roles of SCFAs in host health, individuals with more powerful gut microbiota to utilize Alg and GF could benefit more from *L. japonica* consumption. Thus, for a great number of normal humans, a 14-day supplementation with *L. japonica* could strengthen the capability of gut microbiota to metabolize Alg in *L. japonica*, so to enhance their benefits from *L. japonica* consumption.

### Preferences of Gut Microbiota on the Monosaccharide of GF

**Figure 6:**
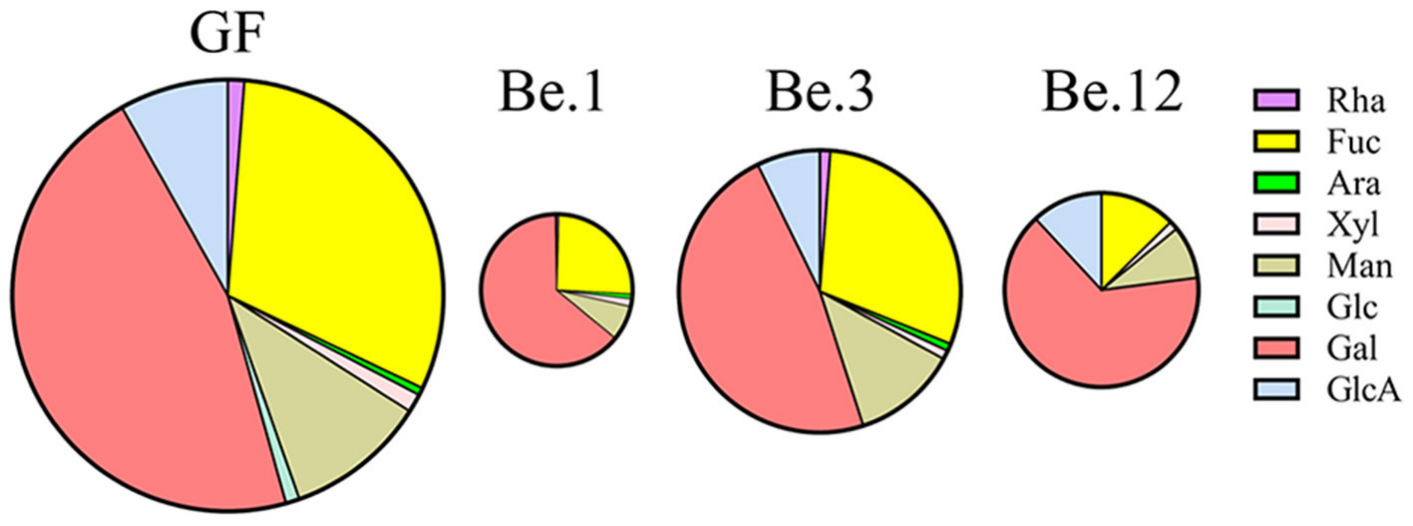
The percentage of monosaccharides in the consumed GF after fermentation by different fecal samples. Be.1, Be.3, and Be.12 were fecal samples obtained from the volunteer Nos. 1, 3, and 12 before the 14-day dietary intake of *L. japonica*, respectively.

It has been well documented that the polysaccharides are metabolized by some CAZymes which are specialized to catalyze certain types of glycosic linkage (49). Due to the substrate specificity of these enzymes in the microbiota, the percentage of monosaccharides in the consumed GF after fermentation could reflect the utilization preferences of gut microbiota. Be.1, Be.3, and Be.12 with higher GF consumption (35.25, 64.54, and 45.09%, respectively) were chosen for monosaccharide composition analysis. As shown in Figure 6, GF was mainly composed of Fuc and Gal with the percentages of 30.83 and 46.18%, respectively. After 48 h of fermentation, the percentage of Gal consumption in Be.1 and Be.12 accounted for 65.79 and 70.23%, larger than that in the original GF. Of note, Be.3, with the most amount of GF consumption, demonstrates a relative ratio of Fuc and Gal similar to that of original GF. The above results demonstrated that Gal residues in GF were more ready to be utilized by intestinal microbiota in Be.1 and Be.12 so as to benefit the growth of beneficial bacteria in the gut. It has been reported that monosaccharides linked to a side chain were more susceptible to being broken down into monomers or dimmers compared with those linked to the backbone (50). It could be hypothesized that Gal residues in GF were possibly linked to the side chains of GF or the glycosidic linkages to Gal were easy to be broken down, resulting in the higher consumption rate of Gal. Whereas, gut microbes of Be.3 may degrade GF by consuming Gal and Fuc residues in GF. The above results indicated that GF could be fermented by specific intestinal microbiota and utilized with a different strategy.

## Conclusion

The present study demonstrated that a 14-day *L. japonica* supplementation could balance *Firmicutes*/*Bacteroidetes* ratio. The Alg utilization capability was significantly enhanced by *L. japonica* supplementation for half of the volunteers. Gut bacteria from *Bacteroidaceae* and *Ruminococcaceae* were positively correlated with Alg utilization while those from *Erysipelotrichaceae, Bacteroidaceae*, and *Prevotellaceae* contributed to the degradation of GF. Of note, the production of acetic acid and the total SCFAs in fermentation were consistent with the consumption of Alg and GF, and the production of propionic acid was positively correlated with the utilization of Alg. Thus, *L. japonica* supplementation could strengthen the Alg utilization so to enhance SCFAs production. In addition, gut microbiota communities showed different monosaccharide preferences in GF consumption. The present study promoted the understanding of the impacts of dietary *L. japonica* on human health.

## Data Availability Statement

The datasets presented in this study can be found in online repositories. The names of the repository/repositories and accession number(s) can be found below: NCBI; PRJNA835688

## Ethics Statement

The studies involving human participants were reviewed and approved by Ethics Committee of Dalian Polytechnic University. The patients/participants provided their written informed consent to participate in this study.

## Author Contributions

XZ: investigation, data curation, and writing-original draft. CS: formal analysis. CC: data curation. GG: methodology. LH: writing-review and editing. ZW: project administration and supervision. SS: conceptualization, writing- original draft, supervision, project administration, and funding acquisition. BZ: project administration. All authors contributed to the article and approved the submitted version.

## Funding

This work was funded by the National Key Research and Development Program of China (No. 2019YFD0902005).

## Conflict of Interest

The authors declare that the research was conducted in the absence of any commercial or financial relationships that could be construed as a potential conflict of interest.

## Publisher's Note

All claims expressed in this article are solely those of the authors and do not necessarily represent those of their affiliated organizations, or those of the publisher, the editors and the reviewers. Any product that may be evaluated in this article, or claim that may be made by its manufacturer, is not guaranteed or endorsed by the publisher.
