# Supplementary material for: Gut Microbiota of Individuals Could Be Balanced by a 14-Day Supplementation With Laminaria japonica and Differed in Metabolizing Alginate and Galactofucan
Source: Front Nutr. 2022 May 18;9:881464. doi: 10.3389/fnut.2022.881464 (PMC9158320; doi:10.3389/fnut.2022.881464)
Supplement: Supplementary file 1 [file Table_1.DOCX]

Supplementary Material

**Table S1.** General characteristics of the twelve volunteers during the intervention.

| Nos. | Gender | Energy intake  (kcal/day) | Be weight  (kg) | Af weight  (kg) | Weight change  (kg) | Height  (cm) | BMI  (kg/m^2^) |
| --- | --- | --- | --- | --- | --- | --- | --- |
| 1 | Man | 2045±177 | 79.10 | 79.10 | 0.00 | 182 | 23.87 |
| 2 | Man | 2057±114 | 69.45 | 68.65 | -0.80 | 175 | 22.67 |
| 3 | Woman | 1400±62 | 52.80 | 51.35 | -1.45 | 168 | 20.25 |
| 4 | Man | 2305±91 | 72.00 | 70.35 | -1.65 | 178 | 22.72 |
| 5 | Man | 1790±87 | 63.30 | 63.50 | 0.20 | 178 | 20.07 |
| 6 | Woman | 1230±98 | 50.40 | 50.90 | 0.50 | 161 | 20.21 |
| 7 | Woman | 1830±110 | 57.80 | 57.75 | -0.05 | 167 | 20.72 |
| 8 | Woman | 1720±51 | 52.89 | 51.35 | -1.54 | 162 | 20.15 |
| 9 | Man | 2159±58 | 60.00 | 59.80 | -0.20 | 178 | 20.83 |
| 10 | Woman | 1430±75 | 50.30 | 50.30 | 0.00 | 165 | 20.31 |
| 11 | Man | 2170±91 | 64.40 | 64.25 | -0.15 | 169 | 22.54 |
| 12 | Woman | 1450±70 | 55.00 | 54.90 | -0.10 | 168 | 19.48 |
